# On the wrong track: ocean acidification attracts larval fish to irrelevant environmental cues

**DOI:** 10.1038/s41598-018-24026-6

**Published:** 2018-04-11

**Authors:** Tullio Rossi, Jennifer C. A. Pistevos, Sean D. Connell, Ivan Nagelkerken

**Affiliations:** Southern Seas Ecology Laboratories, School of Biological Sciences and The Environment Institute, DX 650 418, The University of Adelaide, Adelaide, SA 5005 Australia

## Abstract

Population replenishment of marine life largely depends on successful dispersal of larvae to suitable adult habitat. Ocean acidification alters behavioural responses to physical and chemical cues in marine animals, including the maladaptive deterrence of settlement-stage larval fish to odours of preferred habitat and attraction to odours of non-preferred habitat. However, sensory compensation may allow fish to use alternative settlement cues such as sound. We show that future ocean acidification reverses the attraction of larval fish (barramundi) to their preferred settlement sounds (tropical estuarine mangroves). Instead, acidification instigates an attraction to unfamiliar sounds (temperate rocky reefs) as well as artificially generated sounds (white noise), both of which were ignored by fish living in current day conditions. This finding suggests that by the end of the century, following a business as usual CO_2_ emission scenario, these animals might avoid functional environmental cues and become attracted to cues that provide no adaptive advantage or are potentially deleterious. This maladaptation could disrupt population replenishment of this and other economically important species if animals fail to adapt to elevated CO_2_ conditions.

## Introduction

The process of larval settlement represents a bottleneck in the population dynamics of many marine species. This process is not a simple function of stochastic processes and passive passage via ocean currents. Larval stages often possess sophisticated behaviours, imprinting, and competencies that enable them to identify and navigate towards suitable settlement habitats^1^. Failure to do so would reduce the chance of survival via increased risk of starvation and predation, or settlement in suboptimal habitats. During this critical life history transition, larvae depend on multiple cues such as sounds and odour plumes to orient themselves and progress from a planktonic to a demersal lifestyle^2,3^.

Ocean acidification alters or even reverses many behaviours and cue preferences^4^, by interfering with the processing of sensory information^5^. For example, under future ocean acidification scenarios, clownfish larvae are less attracted to the odour of their settlement habitat and can be attracted to odours of undesirable settlement habitats that are normally ignored or avoided^6^. By far the majority of studies on behaviour and ocean acidification have focussed on vision and olfaction, with relatively little known about its effects on the processing of auditory information. Sound is increasingly recognized as a cue used by dispersing propagules of tropical species because it propagates with little attenuation in water, is independent of currents and also carries information about the habitat type and its resident species community^2,7^. As a result of ocean acidification, larvae of the catadromous barramundi (*Lates calcarifer*) are deterred rather than attracted to the sound of their estuarine settlement habitat at settlement stage^8^, settlement-stage larvae of temperate gobies are deterred by reef soundscapes^9^, whilst post-settlement clownfish lose their avoidance response to reef sounds^10^.

Ocean acidification has the potential to detrimentally impact a range of species by either leading to avoidance of relevant cues, or attraction to irrelevant or misleading cues^6^. Here we test whether ocean acidification can stimulate auditory preferences of fish towards irrelevant or suboptimal cues that would provide no adaptive advantage at settlement stage. In choice experiments, we determined the response of larval barramundi towards soundscapes of habitats located outside of its distribution range, and towards artificially generated white noise as a proxy for irrelevant anthropogenic sounds.

## Results

**Figure 1:**
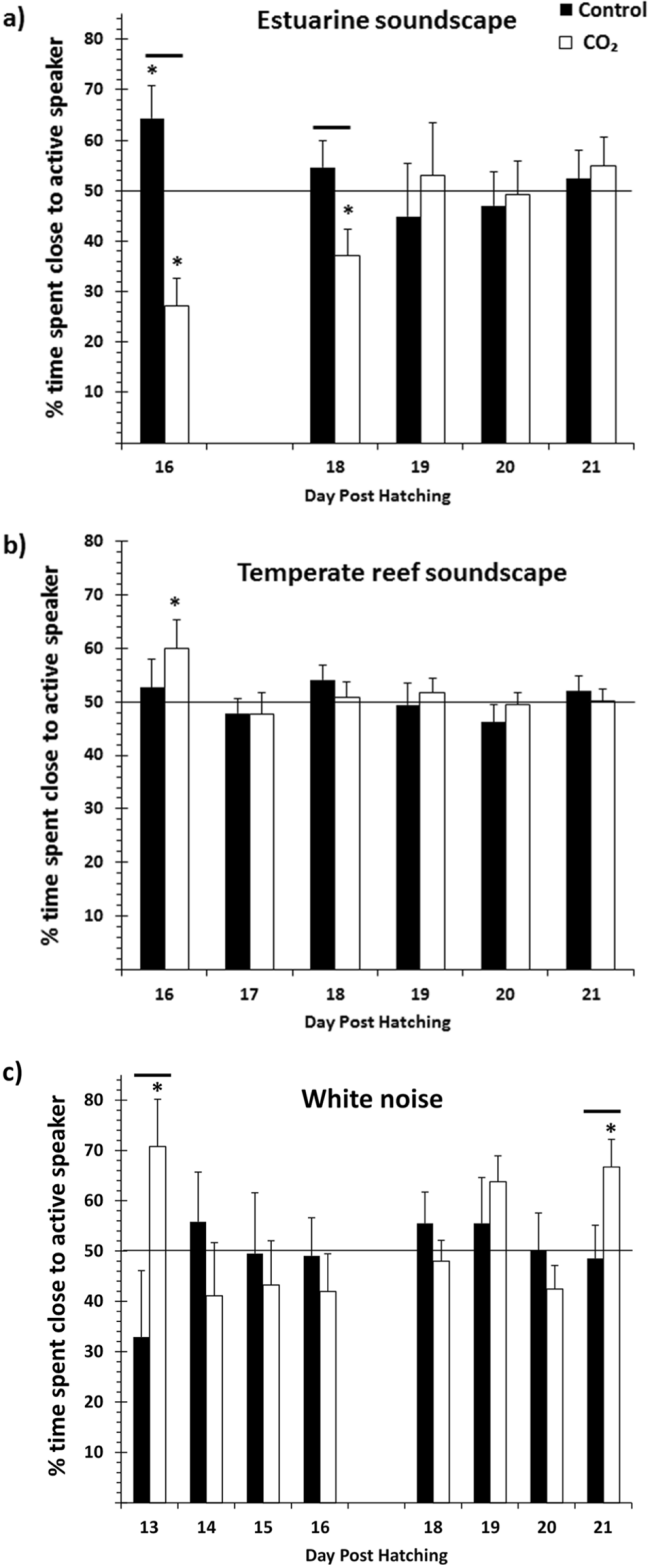
Effect of elevated CO_2_ on preferences for sound cues by barramundi larvae. Average (±SE) percentage of time the larvae were found in the section closest to the active speaker when sound cues were broadcast from: (**a**) settlement habitat (tropical mangrove estuary), (**b**) irrelevant soundscape (temperate reef), and (**c**) artificial noise (white noise). The horizontal lines at 50% indicate lack of any response by larvae (neither deterred nor attracted by the cue). Asterisks indicate a statistically significant difference from a 50% response (Wilcoxon signed rank test). Horizontal lines above bars indicate significant differences (within dph) between control and elevated CO_2_ larvae (2-way ANOVA). N = 10 for control as well as elevated CO_2_ for each day post hatching in Fig. 1a and c. N = 16 for control as well as elevated CO_2_ for each day post hatching in Fig. 1b. Data in (a) are a subset of the data from a previous study^8^ in which larval responses were measured during 13–28 dph but pooled in blocks of 3 days, whilst in the present study only responses for 16–21 dph are used but shown for single days.

Under current-day CO_2_ concentrations, larval barramundi showed a significant (Wilcoxon Signed Rank Test, p = 0.017) attraction to tropical estuarine soundscapes at 16 days post-hatching (dph) (Fig. 1a). As expected, contemporary tropical barramundi larvae showed no response towards either of the two ecologically irrelevant sounds of temperate reefs or artificial white noise (Fig. 1b,c).

However, under projected end-of-century elevated CO_2_ conditions for coastal and estuarine environments, larval barramundi not only showed avoidance towards the soundscape of its natural estuarine settlement habitat (Wilcoxon Signed Rank Test, p = 0.007 on 16 dph and p = 0.028 on 18 dph), but also attraction towards sounds that are ecologically irrelevant and are ignored by present-day larvae. Barramundi raised under elevated CO_2_ showed significant attraction towards temperate rocky reef soundscapes and white noise (Wilcoxon Signed Rank Test, temperate rocky reef: p = 0.043 on 16 dph; white noise: p = 0.047 on 13 dph and p = 0.015 on 21 dph).

Aside from showing a significant attraction or deterrence towards natural and/or irrelevant noises (i.e. signification deviation from a 50% random choice response), larvae raised under present-day conditions also differed in their response from those raised under elevated CO_2_ conditions (Fig. 1): on 16 and 18 dph for natural sounds (2-way ANOVA, CO_2_ treatment × dph interaction: p = 0.006) and on 13 and 21 dph for white noise (CO_2_ treatment × dph interaction: p = 0.037) the % time spent close to the speaker broadcasting sounds differed significantly within dph between larvae raised under the two CO_2_ treatments.

## Discussion

We reveal that ocean acidification not only reduces attraction towards natural habitat cues, but also attracts animals towards ecologically irrelevant auditory cues. We compared the response of larvae towards the sounds of their natural settlement habitat to their response towards sounds from habitats outside of their biogeographic range and to artificially generated white noise. Larvae exposed to ocean acidification were not only deterred to the acoustic cues of their natural settlement habitat, but puzzlingly they were attracted to ecologically irrelevant and potentially misleading sounds. The positive response towards the ecologically irrelevant temperate habitat cues in fish treated with elevated CO_2_ was observed on the same day as when control fish were attracted to tropical estuarine soundscapes. These results are consistent with the only other study that investigated the effect of ocean acidification on the response to both relevant and irrelevant settlement cues^6^. Because that study^6^ focussed on olfactory habitat cues this suggests the existence of a common underlying mechanism that results in altered processing of sensory information regardless of the type of cue (i.e., olfactory or auditory). This is likely due to the fact that elevated CO_2_ concentration causes a reversal in the functioning of the GABA-A receptors from inhibitor to activator which has been shown to alter multiple sensory behaviours in various species of fish^5,11,12^. Hence, ocean acidification has the potential to reverse the natural deterrence of larvae towards ecologically irrelevant cues and attract them to cues and habitats that are not encountered or irrelevant in nature.

The behavioural alterations observed in this study may result in larvae settling in unsuitable habitats or delaying their settlement from the pelagic because of an inability to distinguish relevant from irrelevant environmental cues. Spending longer periods of time in the water column during the larval stage likely incurs elevated mortality, e.g. due to predation or starvation^13^. This risk is likely to be exacerbated where processing of multiple sensory cues, such as sounds and odours, are disrupted simultaneously. Such confounding of cues reduces scope for sensory compensation^14^ or compensation using alternative cues^15^. Because larval settlement into adult habitats is the major bottleneck in the life cycle of many coastal marine species, these results could highlight risk to the replenishment and connectivity of populations of some fish species. Moreover, because ocean acidification has the potential to attract larval fish to anthropogenic sounds such as white noise, this opens up the question whether larvae could also be attracted to other anthropogenic noises commonly found in the ocean such as those produced by boats and ships. As these artificial noises are becoming more and more widespread in our oceans^16^, ocean acidification may add to the already detrimental effects of anthropogenic sounds on species under contemporary climate conditions. We reveal that the effects of ocean acidification on animal behaviours go beyond that of altering responses to natural cues^17^. By reversing behaviour to relevant and irrelevant environmental cues during the critical settlement stage, larval navigation and their population persistence are at risk under future ocean acidification.

## Methods

### Study species

Barramundi is a tropical fish with a distribution ranging from the eastern Indian Ocean to the western Central Pacific, and is of high commercial and recreational importance^15^. Barramundi is a catadromous fish migrating from freshwater to the ocean to spawn. Saltwater (28–35 units) is required for optimal gonadal and larval development^18^. Barramundi eggs and larvae are often caught around river mouths and marine bays, while juveniles settle into mangroves and wetland habitats^18,19^. Our previous experiments on barramundi larvae obtained from the same hatchery (Robarra Broodstock Sanctuary and Hatchery, West Beach, South Australia) as the one for the current experiment, and raised using the same experimental setup, showed that the larvae undergo metamorphosis mostly between the 16 and 21 days post hatching (dph) and are receptive to settlement acoustic cues between the 16 and 18 dph^8^. The larvae settle around 3 weeks post-hatching. Therefore, in this study we tested larvae that were between 16 and 21 days old.

### Larval rearing

Estuaries often have elevated and highly fluctuating *p*CO_2_ concentrations due to natural and human-driven processes like freshwater input, tidal exchange and eutrophication^20,21^. These processes can exacerbate seawater CO_2_ concentrations that are increasing due to greenhouse gas emissions. Projections for end of the century *p*CO_2_ levels in coastal and estuarine range from 1,700 to 3,200 *µ*atm^22^. Because barramundi larvae settle in estuaries and coastal areas we therefore used experimental *p*CO_2_ levels of approx. 1,368 to 1,541 *µ*atm (Table S1) to represent realistic future effects of ocean acidification in estuarine environments.

We used fertilized eggs as well as larvae of 9 dph, depending on the availability of developmental stage at the time of the experiment, both of which were sourced from a local hatchery (Robarra, 7^th^ generation broodstock) and reared at The University of Adelaide. In both cases, two replicate closed-system larval rearing tanks were used for each CO_2_ treatment (i.e. 2 control vs 2 elevated CO_2_ tanks) and comprised individual recirculating tanks consisting of 60 l rearing tanks (containing larvae) connected to their respective 20 l sumps (containing a heater, pump, protein skimmer, UV steriliser, biological filter, and air bubbler) as described in Rossi *et al*.^8^. In summary, CO_2_-enriched air was constantly bubbled into the sumps of the elevated CO_2_-treatments with a Pegas 4000 MF gas mixer (Columbus). The sumps of the ambient treatments were bubbled with ambient air. pH_NBS_ was measured daily within the rearing tanks using a SG2-ELK SevenGo pH probe (Mettler Toledo) calibrated with a three point calibration, whilst salinity was measured daily with a SR6 refractometer (Vital Sine). *p*CO_2_ values of the seawater in the rearing tanks was calculated using the software CO2SYS^23^ with the respective rearing tank values of seawater temperature, salinity, pHNBS and total alkalinity (TA), using constants K1 and K2 from Mehrbach^24^ and refit by Dickson & Millero^25^. Alkalinity was measured twice during each of the two experiments by Dynamic Endpoint Titration using an 888 Titrando (Metrohm) titrator, and values were within 1% accuracy of certified standards (reference materials from Dr. A. Dickson, Scripps Institution of Oceanography). Fish were fed *ad libitum* with rotifers for the first 12 days post hatching (dph), then with *Artemia* nauplii and a dry feed (Otohime) of increasing granule size as development progressed.

We tested the response of larvae to estuarine mangrove sounds and white noise using larvae raised from the egg stage at 1,540 *µ*atm (26 July–14 August 2013), whereas for temperate reef sounds (representing an irrelevant cue from outside the species range) we used larvae raised from 9 days old at 1,360 *µ*atm (5–16 December 2014). CO_2_ treatments (see Table S1) were started at 2 dph for the larvae raised from the egg stage and on 10 dph for the larvae obtained from the hatchery at 9 dph. Four days in elevated CO_2_ treatment were previously reported to be sufficient to trigger behavioural changes in fish^26^. Temperature was maintained at ~ 27 °C (Table S1).

### Effect of elevated CO_2_ on larval fish audition

Three types of recordings were used for the behavioural trials: estuarine soundscapes, which acted as a natural settlement-habitat cue, and two irrelevant cues: temperate reefs and white noise. We used a hydrophone (HiTech HTI-96-MIN with inbuilt preamplifier, manufacturer-calibrated sensitivity −164.3 dB re 1 V/μPa; frequency range 0.02–30 kHz; calibrated by manufacturers; High Tech Inc., Gulfport MS) and digital recorder (2007 recordings: Edirol R-1, 44 kHz sampling rate, Roland Systems Group, Bellingham WA; 2013 recordings: PCM-M10, 48 kHz sampling rate, Sony Inc., Tokyo, Japan) to record the sounds. A recording of mixed estuarine habitats represented a biologically relevant acoustic cue (Figs S1, S2). We randomly mixed 30 sec fragments of mangrove habitat recordings from Tanzania (Kunduchi; obtained between 3:30 and 7 pm during 16–19 Feb. 2007 at 1 m depth during calm seas). The estuarine soundscape data have been published previously but in a different format: in the previous study^8^ larval responses were measured during 13–28 dph but pooled in blocks of 3 days, whilst in the present study only responses for 16–21 dph are used but these are shown for single days rather than pooled days. Soundscapes recorded on temperate rocky reefs in New Zealand (White Island) instead represented an irrelevant biological sound for tropical barramundi as temperate reefs occur outside the biogeographic range of adult habitats. The playback of temperate reef sounds consisted of a 10-minute recording of the dusk chorus, created by mixing various 30 sec fragments collected under full moon between 18–21 November 2013 with a hydrophone placed a few metres above the rocky reef. These recordings were dominated by snapping shrimps crackle, sea urchin raping sounds and the noise of breaking waves. White noise is an artificially generated signal with constant amplitude at every frequency (Figs S1, S2).

Little is known of the hearing sensitivity in barramundi larvae. However, fishes with an extension of their swim bladder to the otic region show increased acoustic sensory performance^27^. Also settlement-stage barramundi are known to have a forward extension of their swim bladder and a gas-filled chamber in the otic region^8^. This enables the transit of sound pressure oscillations from the swim bladder to the area housing the otoliths^28^.

We used the same experimental set-up as designed by Rossi *et al*.^8^. In short, a choice chamber with 8 parallel lanes was used (see Rossi *et al*.^8^ Fig. S5). In each of the 8 lanes, one fish was released at the same time as all the others from the centre of their respective lane (4 control larvae and 4 elevated CO_2_ larvae per trial). Before release, larvae were allowed to acclimate to the experimental conditions for 2 min., during which the respective experimental sound was played for habituation. Upon release, the position of each larvae inside their lane was recorded from above using a camcorder (HF R406 Legria, Canon, Japan). The larvae could choose to swim towards the respective sound broadcast from an underwater speaker (UW-30; maximal output 156 dB re 1 μPa at 1 m, frequency response 0.1–10 kHz, Lubell Labs Inc., Columbus OH) that was located 8 cm from the edge of the chamber. As a control, a second, but silent speaker was located at the other far end of the speaker. The sides of the active vs silent speakers were switched on a daily basis to avoid any effects of tank orientation. Sound could travel inside the lanes as the lanes were separated from the remainder of the tank by mesh. The individual larvae in the 8 lanes are considered true replicates as the fish could not influence each others’ behaviours due to their separation by the PVC sides of the lanes (i.e. they could not hear or smell each other). All tests were done in seawater with ambient *p*CO_2_ levels as altered behavioural effects due to elevated CO_2_ are retained for > 24 hrs when fish arte transferred to ambient conditions^26^. After each trial with 8 fish, we cleaned the tank and chamber and replenished them with unused seawater to get rid of any chemical cues from the previously used fish.

We used EthoVision XT10 (Noldus Information Technology, Wageningen, The Netherlands) to automatically track the position and swimming speed of each larvae for 7 min. after they had been released inside their lane (i.e. after the 2-min habituation). We used this approach to avoid any biases due to observers presence and subjectivity in recording behaviours. Each lane was subdivided in two equal sections and Ethovision recorded the proportion of time each larvae spent in the two sections. The response to each of the three sound cues was investigated daily between 16 and 21 dph, using naïve larvae for each trial (i.e. newly sourced larvae from the rearing tanks). Replication per sound cue per day was: n = 20/day, half in each CO_2_ treatment for larvae raised from the egg stage, and n = 32/day, half in each treatment for larvae raised from 9 dph. No data were collected for the former on day 17 post hatching due to failure of playback equipment. The experiments were all performed from 2–6 pm. Each fish was used only once (i.e. different fish used for different sound experiments). Larvae that did not respond or showed startle behaviour upon release were not included in the statistical analysis (7% of all larvae). For the response to white noise we also tested the responses during 13–15 dph (we were unable to do this for temperate reef sounds). Whilst Rossi *et al*.^8^ showed that barramundi larvae have a very narrow window in which they respond to natural soundscapes (i.e. during 16–18 dph, and not during 13–15 dph or 19–28 dph), we did not have an *a priori* reason to assume this would be the same for responses to anthropogenic noise. Hence, we also investigated the response towards white noise at an earlier stage that the 16–18 dph window, viz. 13–15 dph.

Larvae can continuously sample sound pressure as they approach the sound source (conceptual model provided by Lillis *et al*.^29^) and hence we assumed that larvae inside the lanes could likewise be attracted to sound sources of interest. Besides sound pressure, also particle acceleration is important to consider. We calculated particle acceleration based on the simultaneous measurement of sound pressure by two hydrophones separated by 5.5 cm, and using the Euler equation^30^. Particle acceleration inside the lanes decreased linearly with distance from the active speaker (Fig. S2). Although it is difficult to replicate a far-field acoustic cue in small aquaria^31^, this did not limit our conclusions as we were not interested in determining the exact sensitivity levels of larvae, but were interested in the relative responses (i.e. between CO_2_ treatments) of larvae towards different types of relevant vs irrelevant acoustic cues. The sound pressure levels of the acoustic cues inside the choice chamber was similar to those obtained *in situ* near to the hydrophone, and decreased towards ambient sound levels with increasing distance away from the active speaker^32^ (Figs S1, S2).

### Statistical analysis

Larvae that show no preference toward a sound cue are expected to be randomly distributed inside their lane and hence observed, on average, 50% of the total experimental time in each of the two sections. To test whether larvae spent significantly more than 50% of their time in a particular section (and hence showed a positive response to a particular cue), we used a non-parametric 1-sample Wilcoxon Signed Rank Test. A non-parametric test was used as a Shapiro-Wilk’s test showed that the data did not show a normal distribution. Furthermore, we tested the difference in response between larvae raised on contemporary vs. future CO_2_ conditions within each dph, irrespective of whether they were attracted or deterred towards a sound. This evaluates whether elevated CO_2_ alters the response of larvae towards soundscapes. A 2-way ANOVA was performed for each of the three sounds tested (main factors: CO_2_ treatment and dph), followed by post-hoc comparisons in case of a significant interaction. Response to temperate reef sound showed no significant main effects (treatment: p = 0.529, dph: p = 0.151) or interaction (p = 0.710).

### Data accessibility

Data can be obtained by application to I. Nagelkerken.

### Ethics statement

Research was carried out under approval of the University of Adelaide animal ethics committee (permit: S-2012–171) and according to the University’s animal ethics guidelines.

## Electronic supplementary material

Supplementary information
