# Supplementary material for: On the wrong track: ocean acidification attracts larval fish to irrelevant environmental cues
Source: Sci Rep. 2018 Apr 11;8:5840. doi: 10.1038/s41598-018-24026-6 (PMC5895586; doi:10.1038/s41598-018-24026-6)
Supplement: Supplementary file 1 — Supplementary information [file 41598_2018_24026_MOESM1_ESM.pdf]

On the wrong track: ocean acidification attracts larval fish to irrelevant environmental cues

Tullio Rossi, Jennifer C.A. Pistevos, Sean D. Connell & Ivan Nagelkerken*

Southern Seas Ecology Laboratories, School of Biological Sciences and The Environment Institute, DX 650 418, The University of Adelaide, Adelaide, SA 5005, Australia
*Correspondence and requests for materials should be addressed to I Nagelkerken (ivan.nagelkerken@adelaide.edu.au)

Table S1. Summary of the experimental water chemistry parameters**.**

**Experiment**	**Treatment**	**T(°C)**	**pH NBS**	**N**	**TA (*μ*mol/kg SW)**	***p*CO_2 (*μ*atm)***	**N**	**Salinity**	**N**
Larvae raised from eggs	Control	27.0 (± 0.07)	8.23 (± 0.01)	20	2616 (± 26)	359 (± 22)	2	38.1 (± 0.2)	20
	Elevated	27.0 (± 0.07)	7.73 (± 0.02)	20	2617 (± 21)	1541 (± 20)	2	38.1 (± 0.2)	20
Larvae raised from 9 dph	Control	27.3 (± 0.05)	8.10 (± 0.04)	12	2268 (± 3)	419 (± 38)	3	35.8 (± 0.1)	12
	Elevated	27.4 (± 0.05)	7.71 (± 0.01)	12	2276 (± 7)	1368 (± 32)	3	35.8 (± 0.1)	12

Average (± SE) temperature (T), pH and total alkalinity (TA) measured in the laboratory and their respective sample sizes (N). dph = days post hatching, SW = seawater. * indicates values of *p*CO_2 calculated using CO2SYS.

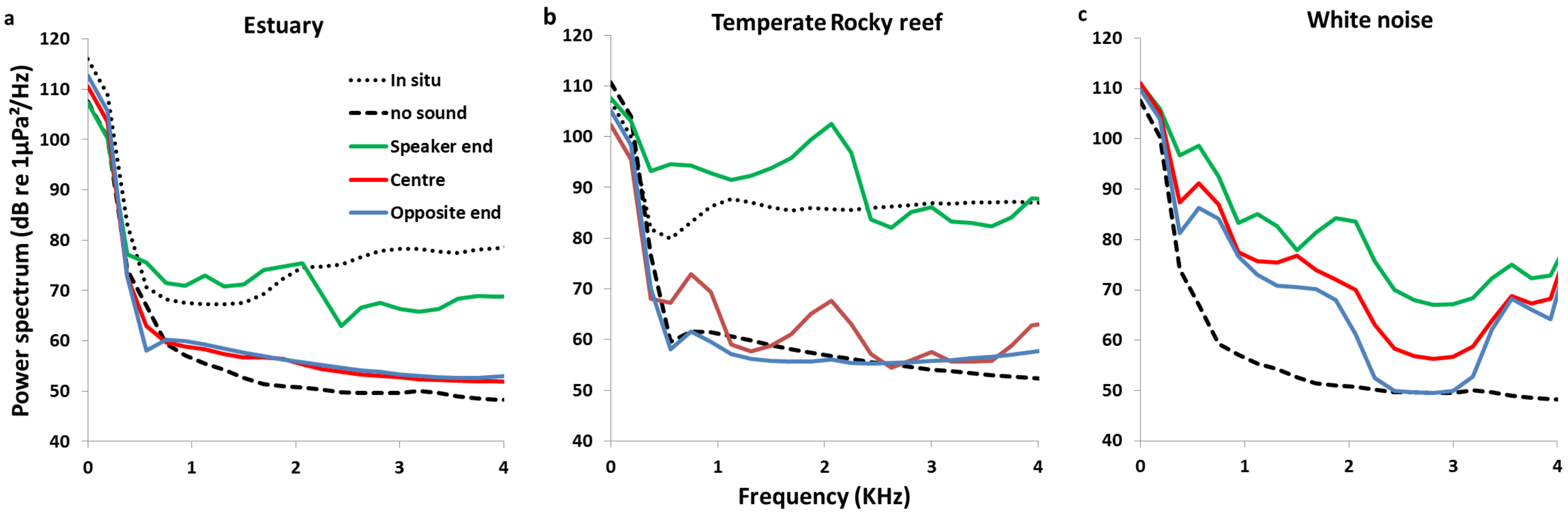

Figure S1. Sound gradients during playback in the choice chamber, showing distinction among the different types of experimental sounds and their relationship to natal habitats (estuaries). Sound pressure gradient from tropical estuarine soundscapes (a), temperate rocky reefs (b) and white noise (b). "Speaker end" represents the part of the choice chamber closest to the speaker, "Centre" represents the middle section of the chamber and "Opposite end" refers to the section of the chamber opposite the speaker. "No sound" refers to the background sound level present in the chamber without playback and "In situ" refers to original field recordings. All measurements were obtained below the water surface and along the centreline of the chamber. Energy below 500 Hz was constant throughout the experiment and originated from noise in the building.

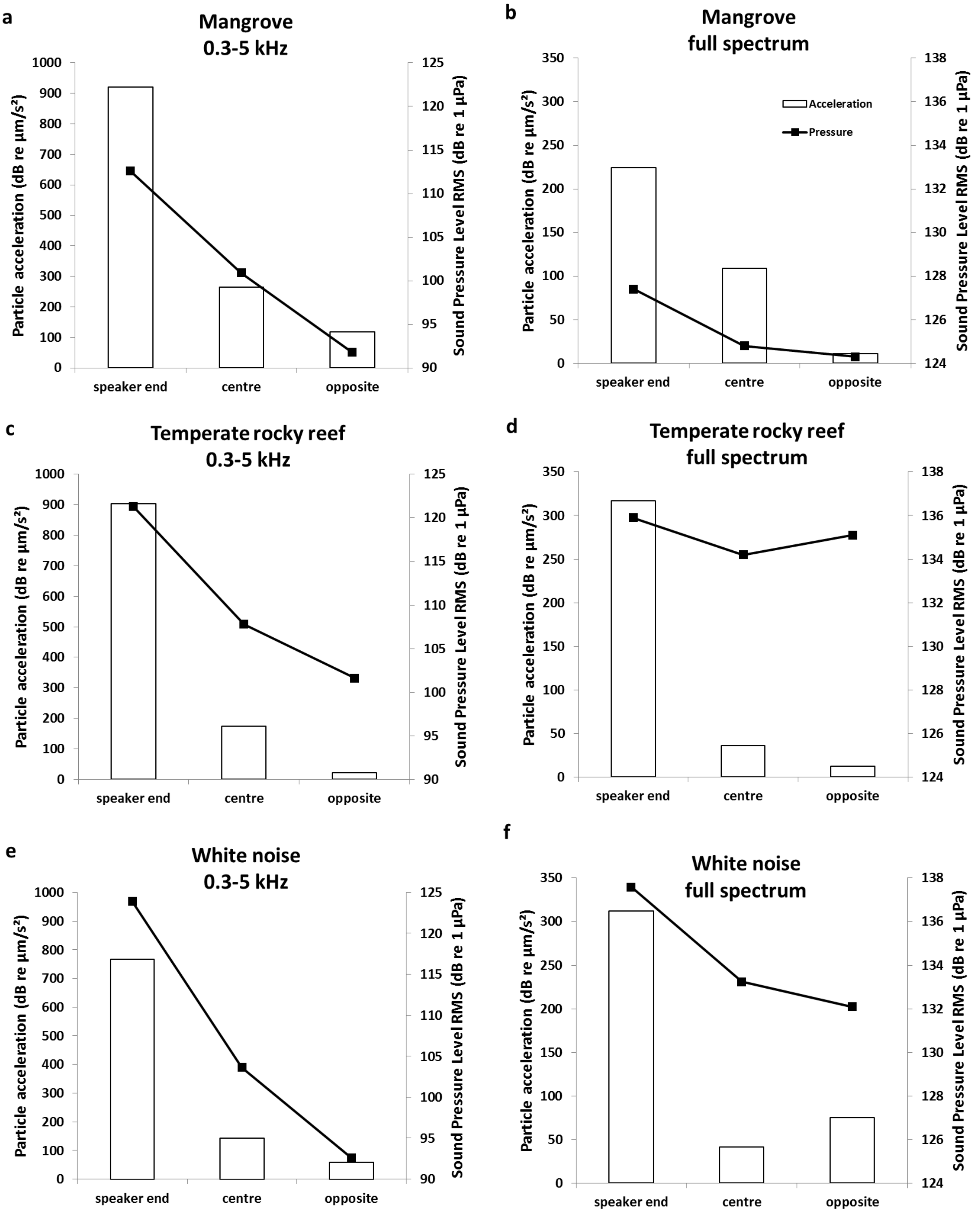

Figure S2. Particle acceleration and sound pressure gradient during playbacks in the experimental choice chamber. (a-c-e) Band pass filtered (0.3-5 kHz) measurements, (c-d-f) full spectrum measurements. (a-b) Tropical estuary (mangrove) soundscape playback, (c-d) temperate reef soundscape, (e-f) white noise. The band pass filter was used to show the sound pressure and particle acceleration patterns in the likely hearing range of fish filtering out higher frequencies (> 5 kHz) and low frequency vibrations in the building (< 0.3 kHz). “Speaker end” represents the part of the choice chamber closest to the speaker, “Centre” represents the middle section of the chamber and “Opposite end” refers to the part of the chamber opposite to the speaker. The sound pressure level in the part of the chamber closest to the speaker was set to match the *in situ* sound pressure level in the hearing range of fish.
